# Dietary casein, egg albumin, and branched-chain amino acids attenuate phosphate-induced renal tubulointerstitial injury in rats

**DOI:** 10.1038/s41598-020-76228-6

**Published:** 2020-11-04

**Authors:** Karin Shimada, Isao Matsui, Kazunori Inoue, Ayumi Matsumoto, Seiichi Yasuda, Yusuke Katsuma, Yusuke Sakaguchi, Minoru Tanaka, Ken Sugimoto, Jun-ya Kaimori, Yoshitsugu Takabatake, Yoshitaka Isaka

**Affiliations:** 1 Department of Nephrology, Osaka University Graduate School of Medicine, 2-2 Yamadaoka, Suita, Osaka 565-0871 Japan; 2 Department of Inter-organ Communication Research in Kidney Disease, Osaka University Graduate School of Medicine, 2-2 Yamadaoka, Suita, Osaka 565-0871 Japan; 3 Department of Geriatric and General Medicine, Osaka University Graduate School of Medicine, Suita, Osaka 565-0871 Japan; 4 Department of Rehabilitation Science, Graduate School of Health Sciences, Kobe University, 7-10-2 Tomoga-oka, Suma, Kobe, Hyogo 654-0142 Japan; 5 Department of Rehabilitation Science, Osaka Health Science University, 1-9-27 Tenma, Kita-ku, Osaka, 530-0043 Japan

**Keywords:** Diseases, Nephrology

## Abstract

Dietary phosphate intake is closely correlated with protein intake. However, the effects of the latter on phosphate-induced organ injuries remain uncertain. Herein, we investigated the effects of low (10.8%), moderate (23.0%), and high (35.2%) dietary casein and egg albumin administration on phosphate-induced organ injuries in rats. The moderate and high casein levels suppressed renal tubulointerstitial fibrosis and maintained mitochondrial integrity in the kidney. The serum creatinine levels were suppressed only in the high casein group. Phosphate-induced muscle weakness was also ameliorated by high dietary casein. The urinary and fecal phosphate levels in the early experiment stage showed that dietary casein did not affect phosphate absorption from the intestine. High dietary egg albumin showed similar kidney protective effects, while the egg albumin effects on muscle weakness were only marginally significant. As the plasma branched-chain amino acid levels were elevated in casein- and egg albumin-fed rats, we analyzed their effects. Dietary supplementation of 10% branched-chain amino acids suppressed phosphate-induced kidney injury and muscle weakness. Although dietary protein restriction is recommended in cases of chronic kidney disease, our findings indicate that the dietary casein, egg albumin, and branched-chain amino acid effects might be reconsidered in the era of a phosphate-enriched diet.

## Introduction

Disturbances in the phosphate metabolism play pivotal roles in the pathogenesis of chronic kidney disease-mineral and bone disorder (CKD-MBD), and the prevalence of hyperphosphatemia increases with CKD progression^1^. As hyperphosphatemia is associated with high mortality, tremendous efforts have been made to establish strategies to lower the phosphate burden^2^. However, the current therapeutic approaches, which are mainly based on dietary phosphate restriction and various phosphate binders (e.g., sevelamer, lanthanum carbonate, bixalomer, sucroferric oxyhydroxide, and ferric citrate), are not satisfactorily effective^3,4^. Therefore, the establishment of novel strategies to protect the organs from phosphate-induced injuries is crucial.

Dietary phosphate intake closely correlates with the protein intake. Based on the published regression equations linking protein to phosphate intakes, the coefficient of determination (*R*^*2*^) has been determined as 0.58–0.84^5,6^. The Japanese Society of Nephrology (JSN), Kidney Disease Outcome Quality Initiative, and Kidney Disease: Improving Global Outcome clinical practice guidelines have recommended dietary protein restrictions of 0.6–0.8, 0.55–0.60, and 0.8 g/kg/day in adults with CKD stages of 3b–5, 3–5, and 4–5, respectively. This intervention for dietary protein in patients with CKD can theoretically benefit nitrogen load reduction and phosphate burden suppression^7–9^. However, considering the recent aging society and the resultant high prevalence of sarcopenia, which can be attenuated by dietary protein, JSN also proposed that dietary protein restriction may be relieved taking a holistic view of individual patients with CKD. Moreover, Shinaberger et al. reported that the risk of controlling the serum phosphate levels by restricting the dietary protein intake may outweigh the benefit of controlled phosphate and could lead to greater mortality; however, the causal relationship remained uncertain in their study^10^. Recently, several interventional studies have demonstrated that dietary protein intake may suppress phosphate burden. Taylor et al. demonstrated that consumption of 225 g of egg whites per day for 6 weeks suppressed the serum phosphate levels in patients subjected to maintenance hemodialysis^11^. Guida et al. showed that replacement of meat and fish with egg white decreased the serum phosphate levels without causing protein malnutrition^12^. These findings suggested that it is meaningful to examine the effects of dietary protein intake on phosphate-induced organ injuries.

Here, we investigated the effects of dietary casein and egg albumin on phosphate-induced organ injuries, especially in the kidney, which plays pivotal roles in the maintenance of phosphate homeostasis^13^. In contrast to the slowly increasing dietary protein consumption worldwide, the consumption of readily absorptive dietary phosphate (i.e., phosphate in processed food and in food additives) has drastically increased over the past half-century^14^. Our results provide novel insights into the dietary intervention for suppressing phosphate toxicity in the era of phosphate-enriched diet.

## Results

### Dietary casein suppressed phosphate-induced renal tubulointerstitial injury

To assess the effects of dietary protein on phosphate-induced kidney injury, the rats were fed experimental diets containing various amounts of casein. Six-week-old male Wistar rats were randomly divided into four groups. Each group received one specific diet, namely normal phosphate (0.9%) + low casein (10.8%) (NPi + LCas group), high phosphate (2.2%) + low casein (HPi + LCas group), high phosphate + moderate casein (23.0%) (HPi + MCas group), and high phosphate + high casein (35.2%) (HPi + HCas group). All the rats were fed the experimental diet ad libitum from the age of 6 to 12 weeks. As CE-2 (i.e., a normal animal diet provided at our animal facility) and American Institute of Nutrition-93G diets contain 25.1% crude protein and 20.0% casein, respectively, we chose to administer 23.0% casein for the HPi + MCas group. The dietary interventions provided in the three high-phosphate groups resulted in increased phosphate excretion in the urine compared to that in the normal phosphate group, at 2 weeks after intervention initiation (*P* < 0.001; Table 1). The urinary and fecal levels of phosphate did not vary among the three high phosphate groups, indicating that phosphate was equally supplemented to the three groups (*P* > 0.05; Table 1). Neither dietary phosphate nor casein affected the fecal calcium levels (*P* > 0.05; Table 1). Dietary phosphate suppressed urinary excretion of calcium, which was restored by high dietary casein (*P* < 0.01 and *P* < 0.05 between the NPi + LCas and HPi + LCas and between the HPi + LCas and HPi + HCas groups, respectively; Table 1).

**Table 1:** Parameters of the casein-, egg albumin-, and phosphate-fed rats.

Parameters	Group NPi + LCas	Group HPi + LCas	Group HPi + MCas	Group HPi + HCas	Group HPi + LAlb	Group HPi + MAlb	Group HPi + HAlb
**Urine (2 w)**							
Phosphate (mg/6 h)	6.2 ± 2.7***	14.6 ± 4.6^Ref^	13.8 ± 4.9^NS^	17.1 ± 2.4^NS^	11.1 ± 2.7^Ref^	11.6 ± 5.8^NS^	8.9 ± 4.7^NS^
Calcium (× 10^–1^ mg/6 h)	1.17 ± 0.90**	0.39 ± 0.12^Ref^	0.49 ± 0.17^NS^	0.91 ± 0.20*	0.55 ± 0.15^Ref^	0.89 ± 0.42^NS^	0.86 ± 0.61^NS^
**Stool (2 w)**							
Phosphate (mg/6 h)	13.9 ± 6.0**	28.0 ± 11.4^Ref^	34.3 ± 6.7^NS^	28.5 ± 6.0^NS^	34.3 ± 10.7^Ref^	40.9 ± 6.9^NS^	42.7 ± 5.0^NS^
Calcium (mg/6 h)	25.7 ± 10.0^NS^	29.2 ± 10.9^Ref^	31.6 ± 7.5^NS^	25.9 ± 5.0^NS^	31.0 ± 11.2^Ref^	39.2 ± 13.7^NS^	39.8 ± 4.9^NS^
**Serum (6 w)**							
Creatinine (mg/dl)	0.62 ± 0.23**	1.11 ± 0.32^Ref^	0.91 ± 0.20^NS^	0.76 ± 0.14*	1.16 ± 0.26^Ref^	1.07 ± 0.12^NS^	0.78 ± 0.21^††^
Urea nitrogen (mg/dl)	14.5 ± 3.6**	32.4 ± 10.6^Ref^	48.9 ± 10.6**	50.8 ± 8.8***	30.4 ± 6.8^Ref^	45.5 ± 13.9^†^	48.3 ± 11.0^††^
Phosphate (mg/dl)	8.8 ± 1.3***	14.5 ± 3.9^Ref^	11.8 ± 2.3^NS^	9.1 ± 1.3***	11.8 ± 3.3^Ref^	9.1 ± 2.5^NS^	8.8 ± 1.5^†^
Calcium (mg/dl)	9.6 ± 0.9**	7.9 ± 0.4^Ref^	8.1 ± 1.0^NS^	7.4 ± 1.2^NS^	8.3 ± 1.0^Ref^	8.5 ± 1.1^NS^	9.5 ± 0.5^†^
**Plasma (6 w)**							
iFGF23 (× 10^3^ pg/ml)	0.36 ± 0.08***	5.5 ± 1.2^Ref^	4.3 ± 1.1^NS^	3.6 ± 1.0**	4.3 ± 2.2^Ref^	1.5 ± 1.4^†^	0.46 ± 0.10^††^
iPTH (pg/ml)	272 ± 306***	2510 ± 532^Ref^	1859 ± 715^NS^	1024 ± 594***	1931 ± 944^Ref^	849 ± 904^NS^	442 ± 331^††^

**Figure 1:**
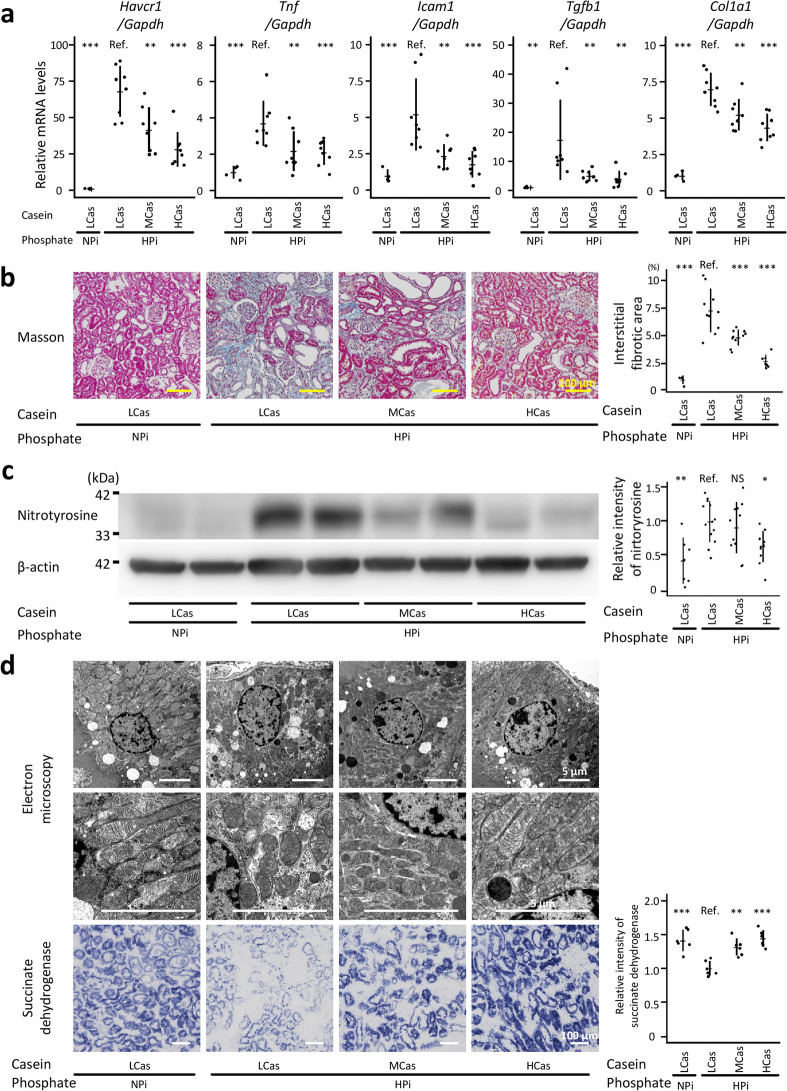
Dietary casein suppressed phosphate-induced renal tubulointerstitial injury. Six-week-old male Wistar rats were randomly divided into the following four groups according to the received diets: NPi + LCas group, normal phosphate (0.9%) + low casein (10.8%); HPi + LCas group, high phosphate (2.2%) + low casein; HPi + MCas group, high phosphate + moderate casein (23.0%); HPi + HCas group, high phosphate + high casein (35.2%). All results were obtained from the rats at 12 weeks of age, except for those of SDH enzymatic staining. The kidney samples for SDH staining were obtained at 7 weeks of age. (**a**) Messenger RNA levels of *Havcr1*, *Tnf*, *Icam1*, *Tgfb1*, and *Col1a1* in the kidney were analyzed with qRT-PCR (N = 4–8 in each group: **P < 0.01, ***P < 0.001, ANOVA followed by Dunnett’s post hoc test). (**b**) Representative micrographs of Masson’s trichrome-stained kidney sections (bars = 100 µm). The interstitial fibrotic area was quantified (N = 4–10 in each group: ***P < 0.001, ANOVA followed by Dunnett’s post hoc test). (**c**) Levels of nitrotyrosine-containing proteins in the kidney were analyzed with western blotting (N = 7–13 in each group: *P < 0.05, **P < 0.01, ANOVA followed by Dunnett’s post hoc test). Full-length blots are presented in Supplementary Fig. S1. Regarding the quantitative analysis, several blots were processed in parallel because the number of gel lanes was not sufficient to simultaneously analyze all the samples in one electrophoresis gel. To enable comparison among parallelly processed blots, several lanes in the parallelly processed blots were applied with the same sample. (**d**) Representative electron micrographs of proximal tubules (bars = 5 µm) and light microscope images of enzymatically stained kidney cortices for SDH activity (bars = 100 µm) (N = 6 in each group: **P < 0.01, ***P < 0.001, ANOVA followed by Dunnett’s post hoc test). All quantitative results are presented as a scatter plot with means ± standard deviations. NS, not significant; Ref, reference; SDH, succinate dehydrogenase.

**Figure 2:**
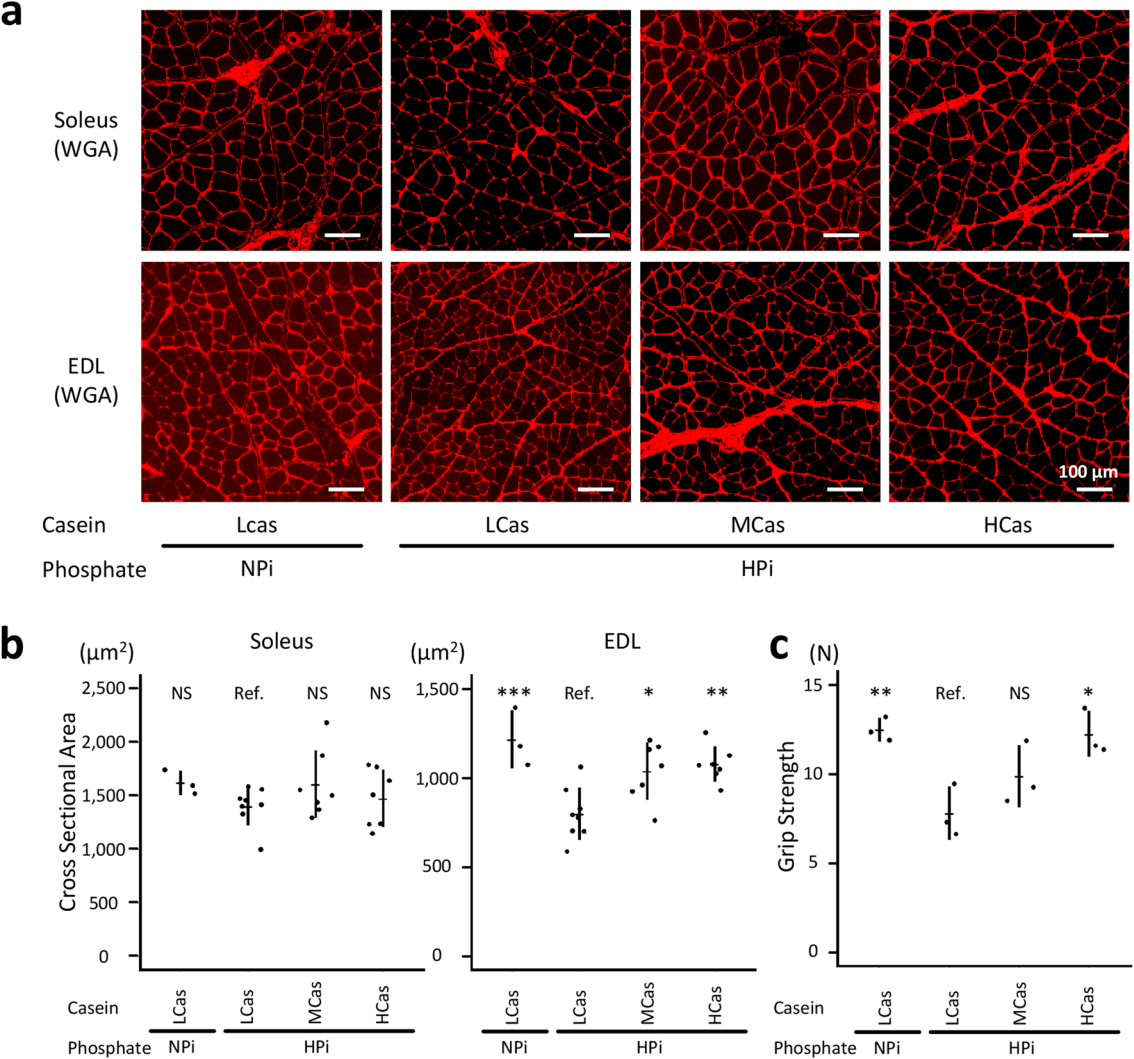
Dietary casein ameliorated phosphate-induced muscular weakness. The rats shown in Fig. 1 at 12 weeks of age were analyzed. (**a**) Representative micrographs of the WGA-stained soleus and EDL muscles are shown (bars = 100 µm). (**b**) Based on WGA-stained sections, cross-sectional area of the muscle fibers was measured (N = 3–8 in each group: *P < 0.05, **P < 0.01, ***P < 0.001, ANOVA followed by Dunnett’s post hoc test). (**c**) The muscular strength was evaluated using the forelimb grip strength test (N = 3 in each group: *P < 0.05, **P < 0.01, ANOVA followed by Dunnett’s post hoc test). All quantitative results are presented as a scatter plot with means ± standard deviations. EDL, extensor digitorum longus; NS, not significant; Ref, reference; WGA, wheat germ agglutinin.

**Figure 3:**
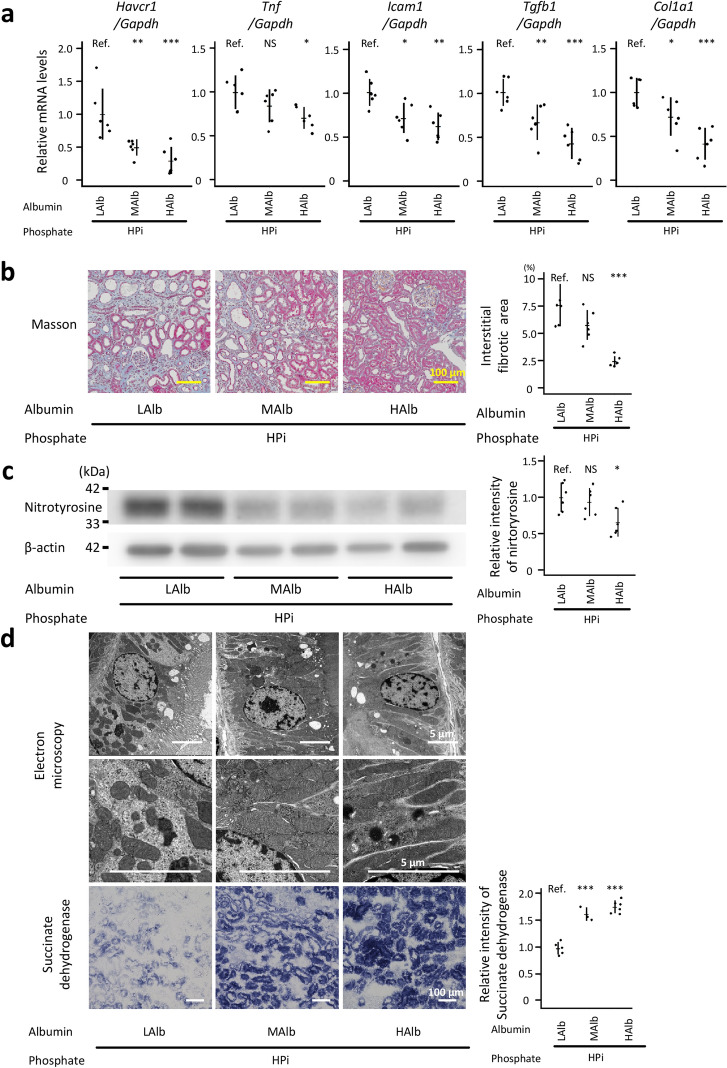
Dietary egg-albumin suppressed phosphate-induced renal tubulointerstitial injury. Six-week-old male Wistar rats were randomly divided into the HPi + LAlb (high phosphate [2.2%] + low egg-albumin [10.8%] diet), HPi + MAlb (high phosphate + moderate egg-albumin [23.0%] diet), and HPi + HAlb (high phosphate + high egg-albumin [35.2%] diet) groups. All results were obtained from the rats at 12 weeks of age, except for those of SDH enzymatic staining. The kidney samples for SDH staining were obtained at 7 weeks of age. (**a**) qRT-PCR analyses for *Havcr1*, *Tnf*, *Icam1*, *Tgfb1*, and *Col1a1* in the kidney are shown (N = 6 in each group: *P < 0.05, **P < 0.01, ***P < 0.001, ANOVA followed by Dunnett’s post hoc test). (**b**) Representative micrographs of Masson’s trichrome-stained kidney sections (bars = 100 µm). Interstitial fibrotic area was quantified (N = 6 in each group: ***P < 0.001, ANOVA followed by Dunnett’s post hoc test). (**c**) Nitrotyrosine-containing protein levels in the kidney were analyzed with western blotting (N = 6 in each group: *P < 0.05, ANOVA followed by Dunnett’s post hoc test). Full-length blots are presented in Supplementary Fig. S2. For the quantitative analysis, several blots were processed in parallel because the number of gel lanes was not sufficient to simultaneously analyze all the samples in one electrophoresis gel. To enable comparison among parallelly processed blots, several lanes in the parallelly processed blots were applied with the same sample. (**d**) Representative electron micrographs of proximal tubules (bars = 5 µm) and light microscope images of enzymatically stained kidney cortices for SDH activity (bars = 100 µm) (N = 3–6 in each group: ***P < 0.001, ANOVA followed by Dunnett’s post hoc test). All quantitative results are presented as a scatter plot with means ± standard deviations. NS, not significant; Ref, reference; SDH, succinate dehydrogenase.

**Figure 4:**
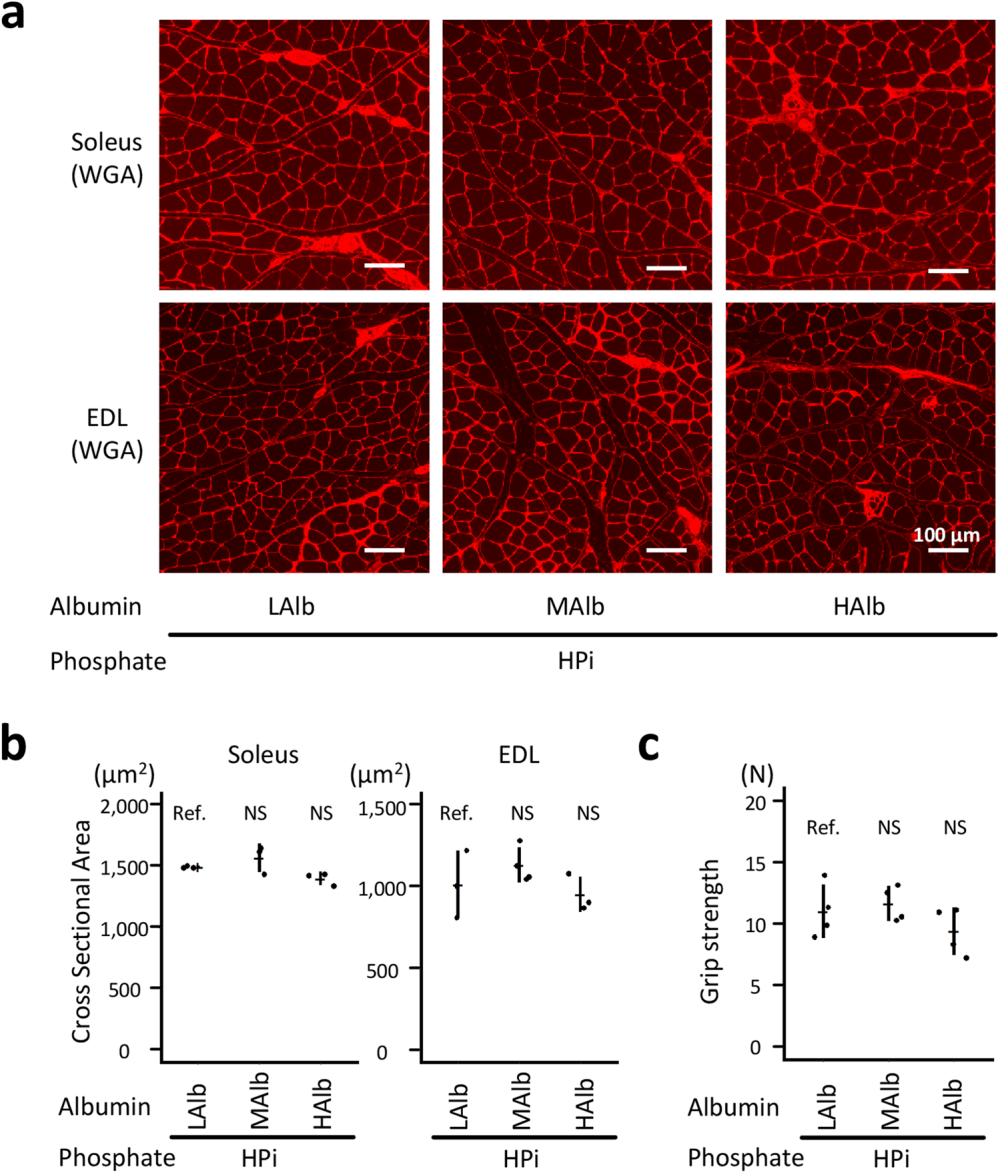
Dietary egg-albumin did not affect phosphate-induced muscular weakness in a dose-dependent manner. The rats shown in Fig. 3 at 12 weeks of age were analyzed. (**a**) Representative micrographs of the WGA-stained soleus and EDL muscles are shown (bars = 100 µm). (**b**) The cross-sectional areas of the muscle fibers were measured (N = 3–4 in each group: ANOVA followed by Dunnett’s post hoc test). (**c**) The muscular strength was evaluated using the forelimb grip strength test (N = 4 in each group: ANOVA followed by Dunnett’s post hoc test). EDL, extensor digitorum longus; WGA, wheat germ agglutinin.

Parameters of the rats shown in Figs. 1, 2, 3 and 4 are summarized. Urine and stool samples were collected at 2 weeks after the initiation of dietary intervention. The serum and plasma samples were collected at the time of dissection. All results are presented as means ± SD. Statistical analysis was performed by ANOVA followed by Dunnett’s post hoc test. The values in the HPi + LCas group served as references for the analyses of the NPi + LCas, HPi + MCas, and HPi + HCas groups (N = 5–10 in each group; *P < 0.05, **P < 0.01, ***P < 0.001, ANOVA followed by Dunnett’s post hoc test). The values of HPi + LAlb group served as references for the analyses of HPi + MAlb and HPi + HAlb groups (N = 5–9 in each group; ^†^*P* < 0.05, ^††^*P* < 0.01, ANOVA followed by Dunnett’s post hoc test).

Abbreviations: iFGF23, intact fibroblast growth factor 23; iPTH, intact parathyroid hormone Ref, reference; NS, not significant; SD, standard deviation; HPi, high phosphate; LCas, low casein; MCas, moderate casein; LAlb, low albumin; MAlb, moderate albumin; HAlb, high albumin; w, week.

High phosphate diets caused renal tubulointerstitial injury under our experimental conditions, and dietary casein suppressed phosphate-induced renal tubulointerstitial injury (Fig. 1 and Table 1). The serum creatine levels significantly increased in the HPi + LCas group than in the NPi + LCas group (*P* < 0.01; Table 1). Quantitative reverse transcription-polymerase chain reaction (qRT-PCR) analyses revealed that the renal mRNA levels for hepatitis A virus cellular receptor 1 *(Havcr1)*, tumor necrosis factor *(Tnf)*, intercellular adhesion molecule 1 *(Icam1)*, transforming growth factor β1 *(Tgfb1)*, and collagen type 1 alpha 1 chain *(Col1a1)* were significantly higher in the HPi + LCas group than in the NPi + LCas group (*P* < 0.001 for *Havcr1, Tnf, Icam1,* and *Col1a1*; *P* < 0.01 for *Tgfb1*; Fig. 1a). Tubulointerstitial fibrosis evaluated using Masson’s trichrome staining demonstrated that a high phosphate diet induced renal tubulointerstitial injury in the HPi + LCas group (*P* < 0.001; Fig. 1b). All the deleterious effects of phosphate on the kidney were ameliorated by high dietary casein (*P* < 0.001 for *Havcr1, Tnf, Icam1, Col1a1,* and *Masson staining*; *P* < 0.01 for *Tgfb1*; *P* < 0.05 for serum creatinine; Fig. 1a,b, and Table 1). Although the serum creatinine levels in the HPi + LCas and HPi + MCas groups were not statistically different, the moderate dietary casein levels also suppressed mRNA expression for *Havcr1*, *Tnf*, *Icam1*, *Tgfb1*, *Col1a1,* and Masson-based fibrosis in the kidney in comparison with those in the HPi + LCas group (*P* < 0.001 for *Masson staining*; *P* < 0.01 for mRNA analyses; *P* > 0.05 for serum creatinine; Fig. 1a,b, and Table 1). Additionally, serum phosphate, plasma intact fibroblast growth factor 23 (iFGF23), and plasma intact parathyroid hormone (iPTH) were suppressed in the HPi + HCas group as compared with those in the HPi + LCas group, although dietary casein did not affect the serum calcium levels (*P* < 0.001 for serum phosphate and plasma iPTH; *P* < 0.01 for plasma iFGF23; *P* > 0.05 for serum calcium; Table 1). Though dietary casein protected the kidney from phosphate-induced injury, the serum urea nitrogen levels were elevated in the HPi + MCas and HPi + HCas groups, reflecting the dietary nitrogen load in these groups (*P* < 0.001 between the HPi + LCas and HPi + HCas groups; *P* < 0.01 between the HPi + LCas and HPi + MCas groups; Table 1). As oxidative stress is an essential mediator of the established renal risk factor pathogenicity, its levels were evaluated in the kidney. The assessment of nitrotyrosine levels (i.e., a modified tyrosine residue formed due to oxidative/nitrative stress) demonstrated that the kidneys of the rats in the HPi + LCas group were exposed to high oxidative stress levels (*P* < 0.01; Fig. 1c and Supplementary Fig. S1). High dietary casein suppressed the nitrotyrosine levels in the kidney (*P* < 0.05; Fig. 1c).

Mitochondria, one of the sources of intracellular reactive oxygen species, are highly dynamic organelles. As mitochondrial morphology, which is continuously reshaped by fission and fusion, is closely associated with the mitochondrial function, the kidney microstructures were analyzed using electron microscopy (Fig. 1d). High phosphate diet was found to promote mitochondrial fission, whereas dietary casein suppressed the fission in the HPi + HCas group (Fig. 1d). Additionally, mitochondrial function assessed with enzymatic histochemical staining of succinate dehydrogenase (SDH) demonstrated that phosphate-induced mitochondrial dysfunction was attenuated in the HPi + MCas and HPi + HCas groups (*P* < 0.001 between the HPi + LCas and NPi + LCas groups and between the HPi + LCas and HPi + HCas groups; *P* < 0.01 between the HPi + LCas and HPi + MCas groups; Fig. 1d).

### Dietary casein ameliorated phosphate-induced muscular weakness

To assess the effects of dietary phosphate and casein on the skeletal muscle, the cross-sectional areas (CSA) of the soleus, which is a representative slow-twitch muscle, and the extensor digitorum longus (EDL), which is a representative fast-twitch muscle, were measured in the rats, as shown in Fig. 1. High phosphate diet reduced the CSA of the EDL, although dietary casein restored it in the HPi + MCas and HPi + HCas groups (*P* < 0.001 between the HPi + LCas and NPi + LCas groups; *P* < 0.01 between the HPi + LCas and HPi + HCas groups; *P* < 0.05 between the HPi + LCas and HPi + MCas groups; Fig. 2a,b). The CSA of the soleus was not affected by phosphate or casein (*P* > 0.05; Fig. 2a,b). Similar to the CSA of the EDL, the grip strength was reduced and restored in the HPi + LCas and HPi + HCas groups, respectively (*P* < 0.01 between the HPi + LCas and NPi + LCas groups; *P* < 0.05 between the HPi + LCas and HPi + HCas groups; *P* > 0.05 between the HPi + LCas and HPi + MCas groups; Fig. 2c).

### Dietary egg albumin protected the kidney from phosphate-induced injury

To examine whether the dietary casein effects could be generalized to the effects of dietary protein, we performed similar experiments, using egg albumin as a dietary source of protein. As casein and egg albumin are the representative heavily- and less-phosphorylated proteins, respectively, performing experiments using egg albumin can reveal whether the property of casein as a phosphoprotein is required to protect the organs from phosphate-induced injuries^15^. Six-week-old male Wistar rats were randomly divided into three groups, and each group received a diet containing high phosphate (2.2%) + low egg albumin (10.8%) (HPi + LAlb group), high phosphate + moderate egg albumin (23.0%) (HPi + MAlb group), or high phosphate + high egg albumin (35.2%) (HPi + HAlb group). The urinary and fecal phosphate and calcium levels were comparable among the three groups, indicating that phosphate was equally supplemented to all the rats under our experimental conditions (*P* > 0.05; Table 1).

All parameters— the mRNA levels of inflammation-related and fibrosis-related genes assessed with qRT-PCR (*P* < 0.001 for *Havcr1, Tgfb1, Col1a1* between the HPi + LAlb and HPi + HAlb groups; *P* < 0.01 for *Icam1* between the HPi + LAlb and HPi + HAlb groups, and for *Havcr1* and *Tgfb1* between the HPi + LAlb and HPi + MAlb groups; *P* < 0.05 for *Tnf* between the HPi + LAlb and HPi + HAlb groups, and for *Icam1* and *Col1a1* between the HPi + LAlb and HPi + MAlb groups; *P* > 0.05 for *Tnf* between the HPi + LAlb and HPi + MAlb groups), tubulointerstitial fibrosis evaluated using Masson’s trichrome-stained kidney sections (*P* < 0.001 between the HPi + LAlb and HPi + HAlb groups; *P* > *0.05* between the HPi + LAlb and HPi + MAlb groups), oxidative stress evaluated by western blotting for nitrotyrosine (*P* < 0.05 between the HPi + LAlb and HPi + HAlb groups; *P* > *0.05* between the HPi + LAlb and HPi + MAlb groups), mitochondrial fission visualized with electron microscopy, mitochondrial function examined with SDH staining (*P* < 0.01), and serum levels of creatinine (*P* < 0.01 between the HPi + LAlb and HPi + HAlb groups; *P* > *0.05* between the HPi + LAlb and HPi + MAlb groups)—revealed that high dietary egg albumin protected the kidney from phosphate-induced tubulointerstitial injury (Fig. 3, Supplementary Fig. S2 and Table 1). The serum phosphate and calcium levels in the HPi + HAlb group were suppressed and elevated, respectively, reflecting an improved kidney function (*P* < 0.05; Table 1). The plasma iFGF23 and iPTH levels were suppressed in the HPi + HAlb group compared with those in the HPi + LAlb group (*P* < 0.01; Table 1). Similar to the results obtained using dietary casein, the serum urea nitrogen levels were elevated after administering dietary egg albumin (*P* < 0.01 between the HPi + LAlb and HPi + HAlb groups; *P* < *0.05* between the HPi + LAlb and HPi + MAlb groups; Table 1).

Additionally, the effects of dietary egg albumin on the muscle were investigated. In contrast to the favorable effects of casein on the muscles, dietary egg albumin did not affect the CSA of the muscular fibers in a dose-dependent manner (*P* > 0.05; Fig. 4a,b). Grip strength was also comparable among the three groups (*P* > 0.05; Fig. 4c). However, even low-dose egg albumin might be effective in protecting the muscle because the difference in grip strength between the HPi + LAlb and HPi + LCas groups was marginally significant (*P* = 0.0817).

### Dietary branched-chain amino acids protected the kidney from phosphate-induced injury

As dietary casein and egg albumin cannot directly act on the kidney, the plasma amino acid levels were measured to investigate how the dietary proteins protect the kidney from tubulointerstitial injuries. Among the amino acids that constitute proteins, only valine, leucine, and isoleucine (branched-chain amino acids [BCAA]) increased in the plasma of casein- and egg albumin-fed rats (valine, *P* < 0.001 between the HPi + LCas and HPi + HCas groups, and between the HPi + LAlb and HPi + HAlb groups; *P* < 0.01 between the HPi + LCas and HPi + MCas groups, and between the HPi + LAlb and HPi + MAlb groups; *P* > 0.05 between the HPi + LCas and NPi + LCas groups; isoleucine, *P* < 0.01 between the HPi + LCas and HPi + MCas groups, and between the HPi + LAlb and HPi + HAlb groups; *P* < 0.05 between the HPi + LCas and HPi + HCas groups, and between the HPi + LAlb and HPi + MAlb groups; *P* > 0.05 between the HPi + LCas and NPi + LCas groups; leucine, *P* < 0.001 between the HPi + LAlb and HPi + MAlb groups, and between the HPi + LAlb and HPi + HAlb groups; *P* < 0.01 between the HPi + LCas and HPi + HCas groups; *P* < 0.05 between the HPi + LCas and HPi + MCas groups; *P* > 0.05 between the HPi + LCas and NPi + LCas groups; Supplementary Tables S1 and S2). Therefore, their effects on phosphate-induced organ injuries were analyzed in a similar manner, as in the experiments shown in Figs. 1, 2, 3 and 4.

To analyze the BCAA effects, we randomly divided the six-week-old male Wistar rats into the HPi + BCAA_0 (high phosphate [2.2%] without BCAA dietary supplementation) and HPi + BCAA_10 (high phosphate with 10.0% BCAA-supplementation diet) groups. The measurement of plasma amino acid levels revealed that the plasma BCAA levels in the HPi + BCAA_10 group did not exceed those in the HPi + HCas and HPi + HAlb groups (*P* < 0.05 in plasma valine values between the HPi + HAlb and HPi + BCAA_10 groups; *P* > 0.05 for all three amino acid levels between the HPi + HCas and HPi + BCAA_10 groups, and for isoleucine and leucine values between the HPi + HAlb and HPi + BCAA_10 groups; Table 2 and Supplementary Tables S1–S3). The urinary and fecal phosphate and calcium levels were not different between the HPi + BCAA_0 and HPi + BCAA_10 groups (*P* > 0.05; Table 3).

**Table 2:** Plasma BCAA levels of the casein-, egg albumin-, or BCAA-fed rats.

Amino acids (nmol/ml)	Group HPi + LCas	Group HPi + HCas	Group HPi + LAlb	Group HPi + HAlb	Group HPi + BCAA_10
Valine	328.4 ± 22.9**	507.8 ± 62.4^NS^	285.0 ± 21.9**	570.3 ± 44.1*	469.4 ± 45.2^Ref^
Isoleucine	173.9 ± 22.2*	221.7 ± 11.9^NS^	150.2 ± 12.0**	255.9 ± 38.4^NS^	221.9 ± 7.0^Ref^
Leucine	299.0 ± 35.1**	401.2 ± 26.7^NS^	239.4 ± 18.9**	411.9 ± 26.1^NS^	400.0 ± 42.2^Ref^

The plasma BCAA levels of the HPi + LCas, HPi + HCas, HPi + LAlb, HPi + HAlb, and HPi + BCAA_10 groups are summarized. All results are presented as means ± SD. The values of the HPi + BCAA_10 group served as references.

Abbreviations: BCAA, branched chain amino acid; Ref, reference; NS, not significant; SD, standard deviation; HPi, high phosphate; LCas, low casein; LAlb, low albumin; MAlb, moderate albumin; HAlb, high albumin; w, week (N = 4–5 in each group; **P < 0.01, ANOVA followed by Dunnett’s post hoc test).

**Table 3:** Parameters of BCAA- and phosphate-fed rats.

Parameters	Group HPi + BCAA_0	Group HPi + BCAA_10
**Urine (2 w)**		
Phosphate (mg/6 h)	17.2 ± 9.1^Ref^	15.3 ± 6.3^NS^
Calcium (mg/6 h) (× 10^–1^ mg/6 h)	0.61 ± 0.26^Ref^	0.74 ± 0.19^NS^
**Stool (2 w)**		
Phosphate (mg/6 h)	43.3 ± 2.4^Ref^	39.8 ± 5.1^NS^
Calcium (mg/6 h)	32.8 ± 9.0^Ref^	37.3 ± 6.7^NS^
**Serum (6 w)**		
Creatinine (mg/dl)	1.08 ± 0.37^Ref^	0.73 ± 0.16*
Urea nitrogen (mg/dl)	30.6 ± 10.1^Ref^	39.3 ± 5.1^NS^
Phosphate (mg/dl)	14.3 ± 4.24^Ref^	8.2 ± 1.2**
Calcium (mg/dl)	7.9 ± 0.4^Ref^	8.6 ± 1.1^NS^
**Plasma (6 w)**		
iFGF23 (pg/ml) (× 10^3^ pg/ml)	4.0 ± 1.4^Ref^	0.87 ± 0.50***
iPTH (pg/ml)	1936.7 ± 797.0^Ref^	700.8 ± 177.2**

**Figure 5:**
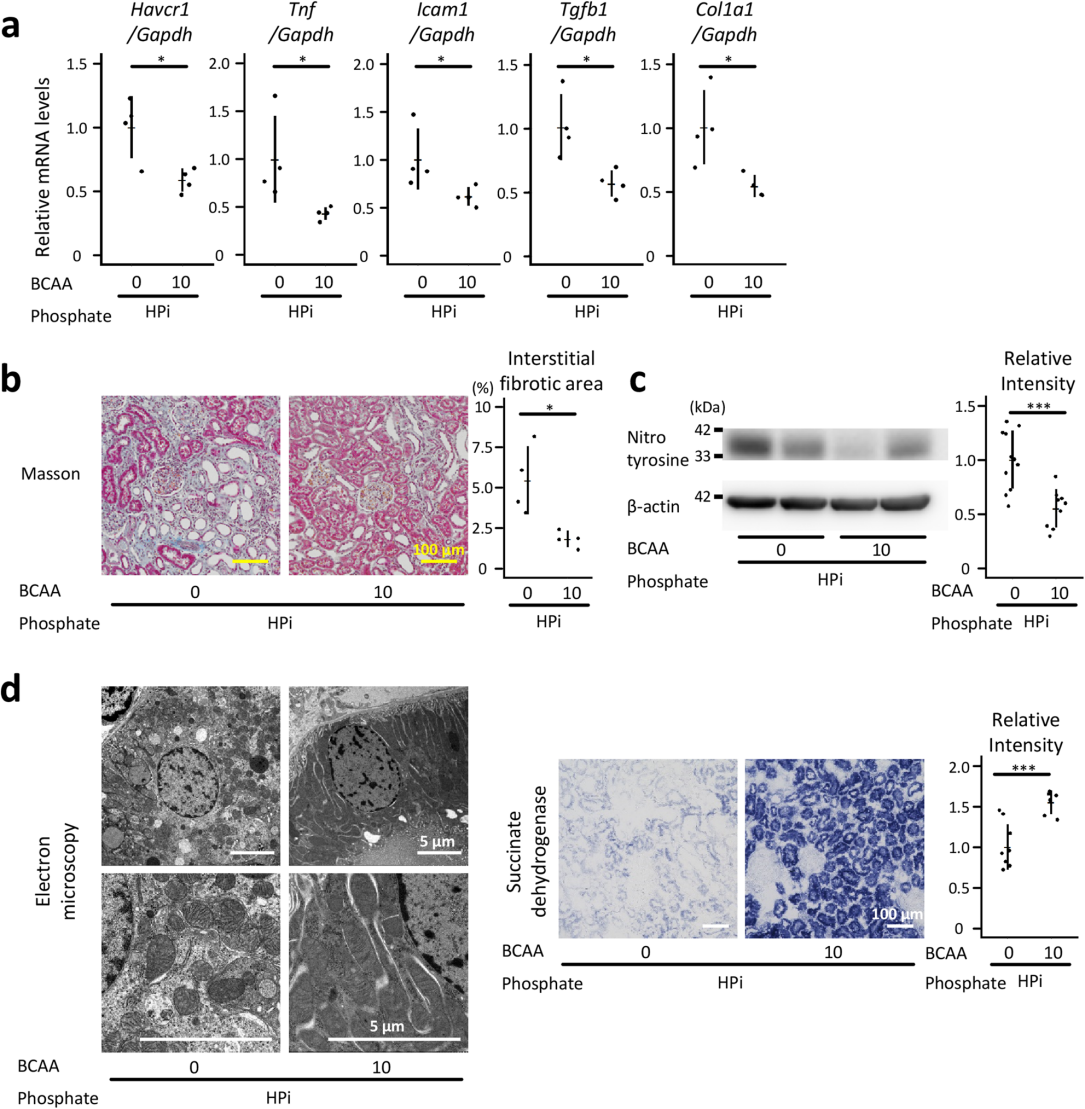
Dietary branched-chain amino acids suppressed phosphate-induced renal tubulointerstitial injury. Six-week-old male Wistar rats were randomly divided into the HPi + BCAA_0 (high phosphate [2.2%] without BCAA-supplemented diet) and HPi + BCAA_10 (high phosphate with BCAA-supplemented [10%] diet) groups. All results were obtained from the rats at 12 weeks of age, except for those of SDH enzymatic staining. The kidney samples for SDH staining were obtained at 7 weeks of age. (**a**) qRT-PCR analyses for *Havcr1*, *Tnf*, *Icam1*, *Tgfb1*, and *Col1a1* in the kidney (N = 4 in each group: *P < 0.05, unpaired t-test). (**b**) Representative micrographs of Masson’s trichrome-stained kidney sections (bars = 100 µm). Interstitial fibrotic area was quantified (N = 4 in each group: *P < 0.05, unpaired t-test). (**c**) Nitrotyrosine-containing protein levels in the kidney were analyzed using western blotting (N = 9–11 in each group: ***P < 0.001, unpaired t-test). Full-length blots are presented in Supplementary Fig. S3. For the quantitative analysis, several blots were processed in parallel because the number of gel lanes was not sufficient to simultaneously analyze all samples in one electrophoresis gel. To enable comparison among parallelly processed blots, several lanes in the parallelly processed blots were applied with the same sample. (**d**) Representative electron micrographs of proximal tubules (bars = 5 µm) and light microscope images of enzymatically stained kidney cortices for SDH activity (bars = 100 µm) (N = 6–9 in each group: ***P < 0.001, unpaired t-test). All quantitative results are presented as a scatter plot with means ± standard deviations. qRT-PCR, quantitative reverse transcription-polymerase chain reaction; SDH, succinate dehydrogenase.

**Figure 6:**
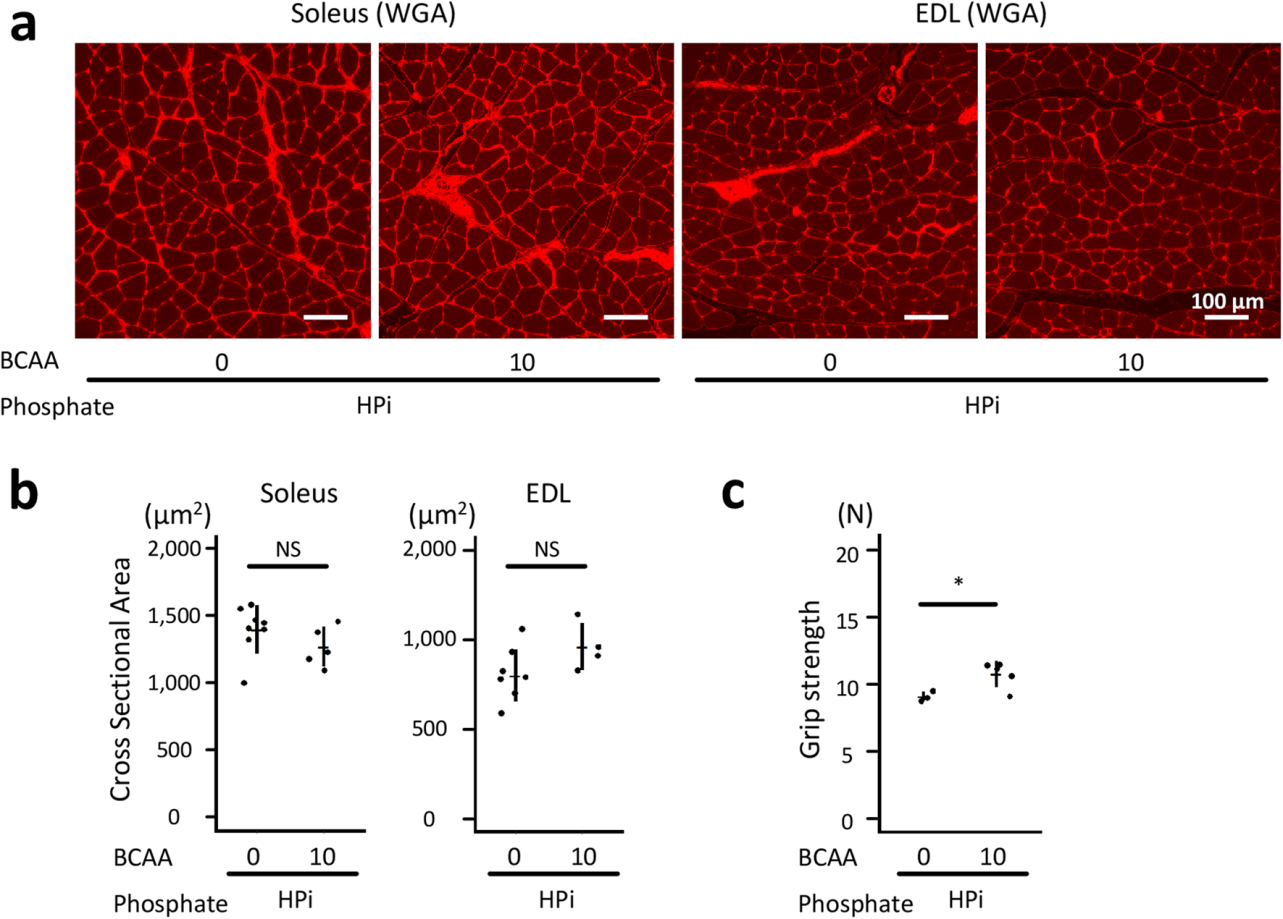
Dietary BCAA suppressed phosphate-induced muscular weakness. The rats shown in Fig. 5 at the age of 12 weeks were analyzed. (**a**) Representative micrographs of the WGA-stained soleus and EDL muscles are shown (bars = 100 µm). (**b**) The cross-sectional areas of the muscle fibers were measured (N = 4–8 in each group: Unpaired t-test). (**c**) The muscular strength was evaluated using the forelimb grip strength test (N = 3–5 in each group: *P < 0.05, Unpaired t-test). EDL, extensor digitorum longus; NS, not significant; WGA, wheat germ agglutinin.

Parameters of the rats shown in Figs. 5 and 6 are summarized. Urine and stool samples were collected at 2 weeks after the initiation of dietary intervention. Serum and plasma samples were collected at the time of dissection. All results are presented as means ± SD. Statistical analysis was performed by the unpaired t-test.

Abbreviations: iFGF23, intact fibroblast growth factor 23; iPTH, intact parathyroid hormone; Ref, reference; NS, not significant; SD, standard deviation; w, week (N = 4–9 in each group; *P < 0.05, **P < 0.01, ***P < 0.001, unpaired t-test).

All analyses—qRT-PCR (*P* < 0.05), Masson’s trichrome staining (*P* < 0.05), western blotting (*P* < 0.001), electron microscopy, SDH staining (*P* < 0.001), and serum creatinine measurements (*P* < 0.05)—showed that BCAA attenuated phosphate-induced renal tubulointerstitial injury (Fig. 5, Supplementary Fig. S3 and Table 3). Corresponding to the improved kidney function, serum phosphate (*P* < 0.01), plasma iFGF23 (*P* < 0.001), and plasma iPTH (*P* < 0.01) were suppressed in the HPi + BCAA_10 group (Table 3). The serum urea nitrogen and calcium levels were not different between the two groups (*P* > 0.05, Table 3). Moreover, BCAA restored grip strength (*P* < 0.05), although the CSA of the muscle was not affected (*P* > 0.05) (Fig. 6).

### Dietary casein, egg albumin, and BCAA restored the levels of protein phosphatase 2C family member (PP2Cm) in the kidney

**Figure 7:**
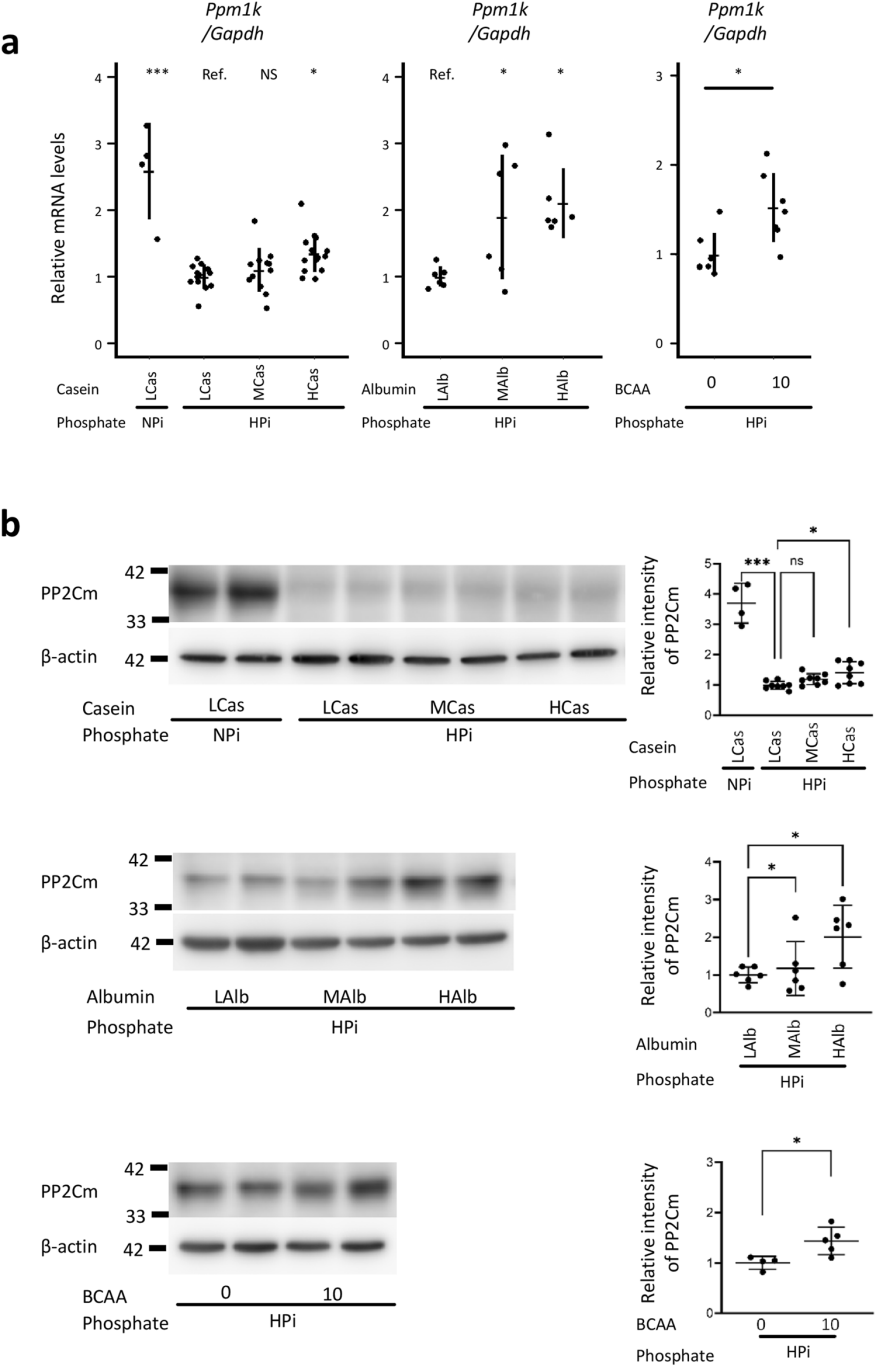
Dietary casein, egg albumin, and BCAA restored the levels of protein phosphatase 2C family member (PP2Cm) in the kidney. The rats shown in Figs. 1, 3, and 5 at 12 weeks of age were analyzed. (**a**) The qRT-PCR analyses for *Ppm1k* in the kidney are presented. For the analyses of casein and egg albumin examinations, ANOVA followed by Dunnett’s post hoc test was performed. For the analysis of BCAA examination, an unpaired t-test was performed (N = 4–13 in each group: *P < 0.05, ***P < 0.001). (**b**) PP2Cm protein levels in the kidney were analyzed with western blotting (N = 4–8 in each group: *P < 0.05, ***P < 0.001). BCAA, branched-chain amino acids; NS, not significant; RT-PCR, quantitative reverse transcription-polymerase chain reaction.

Mitochondria could play pivotal roles in the BCAA catabolism^16^. Excess of BCAA dephosphorylates Ser293 of E1 alpha subunit in the branched-chain-alpha-ketoacid dehydrogenase (BCKD) complex (i.e., the rate-limiting complex in BCAA catabolism), and thereby promotes BCAA degradation^16–18^. PP2Cm, a mitochondrial phosphatase encoded by the *Ppm1k* gene, binds to the BCKD complex and dephosphorylates Ser293 of E1 alpha subunit in the presence of BCKD substrates^17–20^. As several researchers have reported that phosphate at high concentration opens the mitochondrial permeability transition pore (MPTP) (i.e., a non-selective mitochondrial pore that exerts cytotoxic effects), and that mitochondria with PP2Cm-deficiency are highly susceptible to MPTP opening, we examined the *Ppm1k* levels in the kidney^19–22^. Although E1α subunit of BCKD complex is the only substrate identified for PP2Cm to date, it remains uncertain how the defect of PP2Cm regulates MPTP opening^17,23^. Therefore, we analyzed levels mRNA and proteins in the kidney focusing only on *Ppm1k* gene and its product PP2Cm protein. As shown in Fig. 7a, high phosphate diet suppressed the *PPm1k* levels in the kidney (*P* < 0.001). High dietary casein, egg albumin, and 10% BCAA restored the *PPm1k* levels in the kidney (*P* < 0.05 between the HPi + LCas and HPi + HCas groups, and in egg-albumin analyses, and BCAA analysis, *P* > 0.05 between the HPi + LCas and HPi + MCas groups)*.* Western blot analyses also demonstrated that high phosphate diet suppressed PP2Cm levels (*P* < 0.001), while high dietary casein, egg albumin, and 10% BCAA restored PP2Cm levels in the kidney (*P* < 0.05 between the HPi + LCas and HPi + HCas groups, and in egg-albumin analyses, and BCAA analysis, *P* > 0.05 between the HPi + LCas and HPi + MCas groups; Fig. 7b and Supplementary Fig. S4).

## Discussion

In this study, we demonstrated the suppression of phosphate-induced renal tubulointerstitial injury by dietary protein. Dietary casein and egg albumin suppressed the mRNA levels of inflammation- and fibrosis-related genes in the kidney. Additionally, dietary protein maintained the mitochondrial integrity and thereby attenuated oxidative stress in the kidney. Dietary casein and BCAA ameliorated renal tubulointerstitial injury and phosphate-induced muscular weakness. Suppressed plasma iFGF23 and iPTH levels in the HPi + HCas, HPi + HAlb, and HPi + BCAA_10 groups indicated that dietary casein, egg albumin, and BCAA attenuated phosphate burden to the body by suppressing kidney injury. Although in this study we focused on analyzing the effects of dietary protein on phosphate-induced kidney injury and muscular weakness, the target organs for the dietary protein in attenuating phosphate-induced injury are not limited to the kidney and skeletal muscles. Price et al. reported that vascular calcification, promoted by phosphate burden, was detected in only 30% of adenine-induced uremic rats^23^. They speculated that a higher prevalence of vascular calcification may require a longer CKD duration. Based on this, a low-protein diet was designed to reduce the nitrogen load and, thus, to enable the rats to survive on the adenine diet for longer periods. As a result, they unexpectedly found that adenine rats that received a low-protein diet developed extensive vascular calcification without undergoing a long period of feeding^24^. Furthermore, we previously reported that dietary L-lysine (i.e., the first limiting amino acid in most cereal grains) prevents arterial calcification in hyperphosphatemic rats by supporting a proper bone-vascular axis, inhibiting apoptosis of the vascular smooth muscle cells, and attenuating mineral precipitations^25^. Therefore, the consistently reported close correlation between the dietary phosphate and protein levels may represent an intrinsic organ-protective mechanism against phosphate burden.

This study had several limitations. First, the dietary protein and BCAA effects on phosphate-induced organ injuries were analyzed by feeding experimental diets only for a 6-week period. Therefore, the long-term effects and safety of dietary protein remain obscure. Moreover, we measured the urinary and fecal levels of phosphate only at 2 weeks after the initiation of dietary interventions, and not at 6 weeks. Although we showed that casein, egg albumin, and BCAA administration did not affect urinary and stool excretion of phosphate in the HPi + L/M/HCas, HPi + L/M/HAlb, and HPi + BCAA_0/_10 groups, respectively, they might affect excretion of phosphate at 6 weeks after intervention initiation. Second, the effects of amino acids whose plasma levels were suppressed in the HPi + HCas and HPi + HAlb groups were not analyzed. As the plasma levels of alanine, glycine, lysine, serine, and glutamine were suppressed by dietary protein, the reduction in these amino acids might also contribute to organ protection against phosphate-induced toxicity. Third, we cannot rule out the possibilities that small peptides, such as casein phosphopeptides, might play significant roles in the organ protection by dietary casein and egg albumin. Further studies are required to address these issues.

In conclusion, we demonstrated that dietary protein and BCAA protected the kidney and the skeletal muscle against phosphate-induced injuries. Although various phosphate binders are clinically available now, current therapies for hyperphosphatemia are not satisfactory in terms of the pill burden and residual hyperphosphatemia^3,4^. Our results may provide a novel insight for planning an efficient strategy to reduce the phosphate burden.

## Methods

### Animals

Male Wistar rats were purchased from Japan SLC (Hamamatsu, Japan). As shown in Fig. 1, the rats were randomly divided into four groups at 6 weeks of age: the NPi + LCas (normal phosphate [0.9%] + low casein [10.8%] diet), HPi + LCas (high phosphate [2.2%] + low casein diet), HPi + MCas (high phosphate + moderate casein [23.0%] diet), and HPi + HCas (high phosphate + high casein [35.2%] diet) groups. qRT-PCR, Masson’s trichrome-staining, western blot, and SDH-staining analyses were performed using 4–8, 4–10, 7–13, and 6 animals in each group, respectively (Fig. 1). Three to eight animals in each group were used for WGA-staining, while the data of three animals in each group were analyzed to measure the muscle strength (Fig. 2). The parameters shown in the left half portion of Table 1 were measured in 5–10 animals in each group. The plasma amino acid levels were measured in five animals in each group in the casein intervention experiments. As shown in Figs. 3 and 4, the rats were randomly divided into the HPi + LAlb (high phosphate [2.2%] + low egg albumin [10.8%] diet), HPi + MAlb (high phosphate + moderate egg albumin [23.0%] diet), and HPi + HAlb (high phosphate + high egg albumin [35.2%] diet) groups. qRT-PCR, Masson’s trichrome-staining, and western blot analyses were performed using six animals in each group (Fig. 3). SDH-staining was performed using three to six animals. The WGA-stained muscles were analyzed in three to four animals in each group, while the muscular strength was measured in four animals in each group (Fig. 4). The parameters shown in the right half portion of Table 1 were measured in five to nine animals in each group. The plasma amino acid levels were measured in four animals in each group in the egg albumin intervention experiments. To evaluate the dietary BCAA effects on phosphate-induced kidney injury, the rats were randomly divided into the HPi + BCAA_0 (high phosphate [2.2%] without BCAA-supplementation diet) and HPi + BCAA_10 (high phosphate with BCAA-supplementation [10.0%] diet) groups (Figs. 5, 6). qRT-PCR and Masson’s trichrome-staining, western blotting, and SDH staining were performed using 4, 4, 9–11, and 6–9 animals in each group, respectively (Fig. 5). WGA-staining was performed and the muscular strength was measured in four to eight and in three to five animals in each group, respectively (Fig. 6). The parameters shown in Table 3 were measured in four to nine animals in each group. The plasma amino acid levels were measured in four to five animals in each group in the BCAA intervention experiments. In the analyses of *Ppm1k*/PP2Cm, the qRT-PCR data of four to thirteen animals and the western blot data of four to eight animals in each group were analyzed (Fig. 7). All the rats received an experimental diet ad libitum from the age of 6 to 12 weeks. The experimental diet was prepared by modifying the TD05030 diet composition supplied by CLEA Japan (Tokyo, Japan). The compositions of the experimental diet are summarized in Supplementary Tables S4-1, S4-2, S4-3, and S4-4. Casein and starch were purchased from CLEA Japan. Egg albumin, valine, leucine, and isoleucine were purchased from Wako Pure Chemical (Osaka, Japan). The kidney samples for SDH staining were obtained from rats at 7 weeks of age. The urine and stool samples were collected from rats at 8 weeks of age. The samples for other analyses were obtained from rats at 12 weeks of age. All animal experiments were approved by the Animal Committee of Osaka University (approval number 01-046-001), and the study was performed in accordance with standard guidelines regarding the use of animals in scientific experiments.

### Biochemical parameters

The phosphate, calcium and urea nitrogen levels were determined using commercial kits (Wako, Osaka, Japan). The serum creatinine levels were determined using DICT-500 (BioAssay Systems, Hayward, CA, USA). Minerals in the feces were extracted with 150 mM HCl for 3 days at room temperature. The plasma iPTH and iFGF23 levels were measured using the Rat BioActive Intact PTH ELISA (Immutopics, San Clemente, CA, USA) and FGF23 ELISA kits (Kainos, Tokyo, Japan), respectively. The plasma amino acid levels were measured by SRL Co., Ltd (Tachikawa, Japan).

### Histological analyses

Masson’s trichrome (MT) staining was performed using a standard procedure. Digital images of these standard stainings were obtained using a VS120 virtual slide microscope (Olympus, Tokyo, Japan). Fresh cryosections (5-μm thick) were used for SDH enzymatic stain. The sections were incubated for 5 min at 37 °C in a solution mixed with 2 mL nitroblue tetrazolium (NBT) stock (potassium cyanide [6.5 mg/dl], EDTA [185 mg/dl], and NBT [100 mg/dl] in 0.1 M phosphate buffer [pH 7.6] and sodium succinate [500 mM]), 0.2 ml succinate, and 0.7 mg phenazine methosulfate. Wheat germ agglutinin staining of the muscle was performed using a commercial kit (Biotium, Fremont, CA, USA). Muscle fiber images were obtained using BZ-X 800 (Keyence, Osaka, Japan). For electron micrographs, the kidneys were dissected and fixed in 4% glutaraldehyde, post-fixed in 1% osmium tetroxide, and embedded in Epon-Araldite. Images were obtained using an H-7650 transmission electron microscope (Hitachi, Hitachi, Japan). The percentage of blue area in MT-stained section was calculated using ImageJ and Adobe Photoshop (Adobe Systems, San Jose, CA, USA). Randomly selected 10 fields per kidney were used for analyzing the blue area. The relative SDH-stained section intensity was calculated using ImageJ. For the quantification of SDH intensity, five randomly selected cortex fields per kidney were analyzed. The CSA was calculated using a BZ-X analyzer (Keyence, Osaka, Japan). At least 100 muscle fibers per animal were randomly selected for calculating CSA^26^.

### Quantitative reverse transcription-PCR

To quantify the mRNA expression levels, qRT SYBR-Green PCR analysis was performed using ABI PRISM 7900HT (Applied Biosystems, Foster City CA, USA). The primer sets are shown in Supplementary Table S5. RNA was extracted from the whole kidney tissues with TRIZOL (Invitrogen, Carlsbad, CA, USA) according to the manufacturer’s instructions.

### Western blot analyses

Nitrotyrosine (ab 7048; Abcam, Oregon, USA) , PP2Cm (ab 135,286; Abcam, Oregon, USA) and β-actin (A5441; Sigma-Aldrich, St Louis, MO, USA) antibodies were purchased. The dilution rates of the primary antibodies were 1:2,000 for nitrotyrosine and PP2Cm, and 1:7,000 for β-actin. Western blot analyses were performed, as described previously^27–29^.

### Measurement of the grip strength

Forelimb grip strength measurement was conducted, as previously described, using a Grip Strength Meter (Muromachi Kikai, Tokyo, Japan)^30^. Each rat was kept horizontally and pulled steadily until its grip was released. Grip strength was measured twice in each rat.

### Statistical analysis

Comparisons between two groups and among multiple groups were performed using the unpaired t-test and ANOVA followed by Dunnett’s post hoc test, respectively. Statistical significance was defined as *P* < 0.05. All data were statistically analyzed using JMP Pro 11.1.1 for Windows (SAS Institute, Cary, NC, USA).

## Supplementary information

Supplementary information.

## Data Availability

The datasets generated during and/or analyzed during the current study are available from the corresponding author on reasonable request.
