# Supplementary material for: Dietary casein, egg albumin, and branched-chain amino acids attenuate phosphate-induced renal tubulointerstitial injury in rats
Source: Sci Rep. 2020 Nov 4;10:19038. doi: 10.1038/s41598-020-76228-6 (PMC7643071; doi:10.1038/s41598-020-76228-6)
Supplement: Supplementary file 1 — Supplementary information. [file 41598_2020_76228_MOESM1_ESM.docx]

**Dietary casein, egg albumin, and branched-chain amino acids attenuate phosphate-induced renal tubulointerstitial injury in rats**

Karin Shimada, MD^1^, Isao Matsui, MD, PhD^1*^, Kazunori Inoue, MD, PhD^1^, Ayumi Matsumoto, MD^1^, Seiichi Yasuda, MD^1^, Yusuke Katsuma, MD^1^, Yusuke Sakaguchi, MD, PhD^2^, Minoru Tanaka, PhD^3,4,5^, Ken Sugimoto, MD, PhD^3^, Jun-ya Kaimori, MD, PhD^2^, Yoshitsugu Takabatake, MD, PhD^1^, Yoshitaka Isaka, MD, PhD^1^

**Supplementary Material**

Supplementary Table S1. Plasma amino acids levels of casein-fed rats

Supplementary Table S2. Plasma amino acids levels of egg albumin-fed rats

Supplementary Table S3. Plasma amino acids levels of BCAA-fed rats

Supplementary Table S4-1. Composition of the casein/starch free TD05030 diet

Supplementary Table S4-2. Composition of the experimental diet (casein diet)

Supplementary Table S4-3. Composition of the experimental diet (egg albumin diet)

Supplementary Table S4-4. Composition of the experimental diet (BCAA diet)

Supplementary Table S5. Primer nucleotide sequences

Supplementary Figure S1. Full blot of Fig. 1c

Supplementary Figure S2. Full blot of Fig. 3c

Supplementary Figure S3. Full blot of Fig. 5c

Supplementary Figure S4. Full blot of Fig. 7b

**Supplementary Table S1. Plasma amino acids levels of casein-fed rats**

| Amino Acids (nmol/ml) | Group  NPi + LCas | Group  HPi + LCas | Group  HPi + MCas | Group  HPi + HCas |
| --- | --- | --- | --- | --- |
| Alanine | 524.4±193.2 * | 823.8±149.0 ^Ref^ | 623.7±153.9 ^NS^ | 432.9±34.5 ** |
| Glycine | 122.6±17.7 *** | 199.6±34.8 ^Ref^ | 133.4±24.9 ** | 99.2±13.8 *** |
| Valine | 374.3±61.4 ^NS^ | 328.4±22.9 ^Ref^ | 460.9±39.0 ** | 507.8±62.4 *** |
| Isoleucine | 191.4±31.6 ^NS^ | 173.9±22.2 ^Ref^ | 226.5±21.5 ** | 221.7±11.9 * |
| Leucine | 341.7±67.0 ^NS^ | 299±35.1 ^Ref^ | 380.2±42.8 * | 401.2±26.7 ** |
| Phenylalanine | 69.6±3.9 ^NS^ | 86.6±17.5 ^Ref^ | 78.1±7.1 ^NS^ | 72.0±5.8 ^NS^ |
| Tyrosine | 140.1±49.9 ^NS^ | 133±15.7 ^Ref^ | 111.8±11.6 ^NS^ | 112.2±13.2 ^NS^ |
| Tryptophan | 101.0±15.1 ^NS^ | 104.2±16.8 ^Ref^ | 119.6±11.9 ^NS^ | 125.9±7.4 ^NS^ |
| Methionine | 54.2±11.4 ^NS^ | 53.0±3.4 ^Ref^ | 56.1±6.9 ^NS^ | 50.1±5.1 ^NS^ |
| Cystine | 19.8±14.2 ^NS^ | 10.4±3.9 ^Ref^ | 12.9±3.7 ^NS^ | 15.6±4.2 ^NS^ |
| Proline | 271.0±116.4 ^NS^ | 419.5±49.6 ^Ref^ | 478.1±120.1 ^NS^ | 363.1±119.6 ^NS^ |
| Lysine | 562.6±108.9 ^NS^ | 598.7±19.9 ^Ref^ | 449.3±81.0 * | 404.7±81.2 ** |
| Arginine | 158.3±26.8 ^NS^ | 155.5±14.6 ^Ref^ | 129.3±4.1 * | 119.3±11.8 ** |
| Histidine | 114.7±16.8 ^NS^ | 134.1±13.8 ^Ref^ | 104.6±12.4 ** | 85.8±9.6 *** |
| Aspartic acid | 8.2±2.3 ** | 11.9±1.7 ^Ref^ | 8.0±1.4 ** | 8.2±0.7 ** |
| Glutamic acid | 63.2±13.2 * | 95.7±18.7 ^Ref^ | 78.7±14.8 ^NS^ | 83.8±16.7 ^NS^ |
| Serine | 435.3±40.1 *** | 614.4±72.5 ^Ref^ | 278.6±47.7 *** | 212.2±19.4 *** |
| Threonine | 389.5±51.7 ** | 601.3±132.7 ^Ref^ | 559.0±72.0 ^NS^ | 372.6±58 ** |
| Asparagine | 72.2±16.7 ^NS^ | 76.2±7.8 ^Ref^ | 74.6±16.5 ^NS^ | 64.5±9.7 ^NS^ |
| Glutamine | 796.7±81.0 ^NS^ | 828.6±41.6 ^Ref^ | 677.6±56 ** | 548.6±51.0 *** |

Plasma amino acid levels of the rats shown in Fig. 1-2 are summarized. Plasma samples were collected at the time of dissection. All results are presented as mean ± SD values. Statistical significance was evaluated by ANOVA followed by Dunnett’s post hoc test. Group HPi + LCas served as references. Abbreviations: Ref, reference; NS, not significant. (N = 5 in each group; **P* < 0.05, ***P* < 0.01, ****P* < 0.001, ANOVA followed by Dunnett’s post hoc test)

**Supplementary Table S2. Plasma amino acids levels of egg albumin-fed rats**

| Amino Acids (nmol/ml) | Group  HPi + LAlb | Group  HPi + MAlb | Group  HPi + HAlb |
| --- | --- | --- | --- |
| Alanine | 610.8±112.7 ^Ref^ | 489.4±369.7 ^NS^ | 286.2±59.4 ^NS^ |
| Glycine | 200.2±50.8 ^Ref^ | 156.6±40.2 ^NS^ | 106.0±18.1 * |
| Valine | 285±21.9 ^Ref^ | 447.8±61.2 ** | 570.3±44.1 *** |
| Isoleucine | 150.2±12.0 ^Ref^ | 208.1±34.0 * | 255.9±38.4 ** |
| Leucine | 239.4±18.9 ^Ref^ | 346.2±31.9 *** | 411.9±26.1 *** |
| Phenylalanine | 72.2±3.1 ^Ref^ | 80.2±12.2 ^NS^ | 74.4±8.0 ^NS^ |
| Tyrosine | 98.1±18.5 ^Ref^ | 126.8±38.7 ^NS^ | 114.3±13.5 ^NS^ |
| Tryptophan | 77.6±13.9 ^Ref^ | 117.1±18.0 ** | 125.3±13.1 ** |
| Methionine | 47.7±3.6 ^Ref^ | 57.9±25.3 ^NS^ | 48.1±12.3 ^NS^ |
| Cystine | 14.1±3.6 ^Ref^ | 16.7±3.4 ^NS^ | 15.8±3.0 ^NS^ |
| Proline | 182.8±11.9 ^Ref^ | 162.3±85.4 ^NS^ | 115.9±24.6 ^NS^ |
| Lysine | 530.4±85.1 ^Ref^ | 425.7±94.0 ^NS^ | 372.8±57.5 * |
| Arginine | 176.3±26.0 ^Ref^ | 162.1±39.3 ^NS^ | 127.9±21.4 ^NS^ |
| Histidine | 83.5±8.7 ^Ref^ | 86.7±15.6 ^NS^ | 76.3±1.5 ^NS^ |
| Aspartic acid | 7.7±0.8 ^Ref^ | 12.1±10.8 ^NS^ | 7.8±1.9 ^NS^ |
| Glutamic acid | 65.0±7.4 ^Ref^ | 74.0±29.2 ^NS^ | 61.0±10.3 ^NS^ |
| Serine | 368.2±43.5 ^Ref^ | 221.9±55.5 ** | 170.9±21.6 *** |
| Threonine | 165.9±33.9 ^Ref^ | 323.5±71.0 ** | 236.0±43.8 ^NS^ |
| Asparagine | 59.4±9.5 ^Ref^ | 56.6±23.6 ^NS^ | 45.9±11.3 ^NS^ |
| Glutamine | 698.2±41.8 ^Ref^ | 497.7±50.6 *** | 429.4±47.1 *** |

Plasma amino acid levels of the rats shown in Fig. 3-4 are summarized. Plasma samples were collected at the time of dissection. All results are presented as mean ± SD values. Statistical significance was evaluated by ANOVA followed by Dunnett’s post hoc test. Group HPi + LAlb served as references. Abbreviations: Ref, reference; NS, not significant. (N = 4 in each group; **P* < 0.05, ***P* < 0.01, ****P* < 0.001, ANOVA followed by Dunnett’s post hoc test)

**Supplementary Table S3. Plasma amino acids levels of BCAA-fed rats**

| Amino Acids (nmol/ml) | Group  HPi + BCAA_0 | Group  HPi + BCAA_10 |
| --- | --- | --- |
| Alanine | 648.7±161.5 ^Ref^ | 542.8±124.4 ^NS^ |
| Glycine | 175.6±44.6 ^Ref^ | 85.4±19.8 ** |
| Valine | 343.1±18.0 ^Ref^ | 469.4±45.2 *** |
| Isoleucine | 171.9±5.1 ^Ref^ | 221.9±7.0 *** |
| Leucine | 290.4±21.1 ^Ref^ | 400±42.2 ** |
| Phenylalanine | 77.2±9.1 ^Ref^ | 75.7±7.0 ^NS^ |
| Tyrosine | 145.5±26.6 ^Ref^ | 115.8±6.1 ^NS^ |
| Tryptophan | 96.9±17.3 ^Ref^ | 112.4±12.3 ^NS^ |
| Methionine | 56.3±7.3 ^Ref^ | 63.3±10.3 ^NS^ |
| Cystine | 15.8±6.5 ^Ref^ | 16.6±4.3 ^NS^ |
| Proline | 361.3±100.7 ^Ref^ | 261.8±40.6 ^NS^ |
| Lysine | 561.5±85.7 ^Ref^ | 561.9±89.2 ^NS^ |
| Arginine | 155±17.3 ^Ref^ | 157.9±15.5 ^NS^ |
| Histidine | 127.1±18.5 ^Ref^ | 96.9±13.7 * |
| Aspartic acid | 10.9±1.8 ^Ref^ | 8.1±4.0 ^NS^ |
| Glutamic acid | 97.7±21.0 ^Ref^ | 76.2±15.6 ^NS^ |
| Serine | 532.4±99.0 ^Ref^ | 299.1±41.3 ** |
| Threonine | 539.3±185.7 ^Ref^ | 214.5±25.9 * |
| Asparagine | 73.1±11.1 ^Ref^ | 65.5±9.2 ^NS^ |
| Glutamine | 787.5±79.9 ^Ref^ | 932.8±59.1 * |

Plasma amino acid levels of the rats shown in Fig. 5-6 are summarized. Plasma samples were collected at the time of dissection. All results are presented as mean ± SD values. Statistical significance was evaluated by unpaired t test. Abbreviations: Ref, reference; NS, not significant. (N = 4-5 in each group; **P* < 0.05, ***P* < 0.01, ****P* < 0.001, unpaired t test)

**Supplementary Table S4-1. Composition of the casein/starch free TD05030 diet**

| Composition (%) |  |
| --- | --- |
| Casein | 0 |
| Starch | 0 |
| Maltodextrin | 22.6837 |
| Granulated sugar | 43.8981 |
| Crystalline cellulose | 8.7245 |
| Soybean oil | 8.0265 |
| Mineral mix (AIN-93G) | 6.1071 |
| Vitamin mix (AIN-93VX) | 1.7449 |
| Choline bitartrate | 0.4362 |
| L-cystine | 0.0698 |
| Tertiary butylhydroquinone | 0.0017 |
| Ca(H_2_PO_4_)_2_・H_2_O | 5.2870 |
| K_3_C_6_H_5_O_7_・H_2_O | 1.3959 |
| MgO | 0.3490 |
| CaCO_3_ | 0.3420 |
| NaCl | 0.9336 |

**Supplementary Table S4-2. Composition of the experimental diet (casein diet)**

| Composition (%) | Group  NPi  + LCas | Group  HPi  + LCas | Group  HPi  + MCas | Group  HPi  + HCas |
| --- | --- | --- | --- | --- |
| Casein | 10.8 | 10.8 | 23.0 | 35.2 |
| Egg albumin | 0 | 0 | 0 | 0 |
| BCAA | 0 | 0 | 0 | 0 |
| Starch | 29.2 | 26.4 | 14.2 | 2.0 |
| KH_2_PO_4_ | 0 | 5.6 | 5.6 | 5.6 |
| TD05030 | 60.0 | 57.2 | 57.2 | 57.2 |

Composition of the experimental diet used in Fig. 1-2 is summarized. Abbreviations: BCAA, branched-chain amino acid.

**Supplementary Table S4-3. Composition of the experimental diet (egg albumin diet)**

| Composition (%) | Group  HPi  + LAlb | Group  HPi  + MAlb | Group  HPi  + HAlb |
| --- | --- | --- | --- |
| Casein | 0 | 0 | 0 |
| Egg albumin | 10.8 | 23.0 | 35.2 |
| BCAA | 0 | 0 | 0 |
| Starch | 26.4 | 14.2 | 2.0 |
| KH_2_PO_4_ | 5.6 | 5.6 | 5.6 |
| TD05030 | 60.0 | 60.0 | 60.0 |

Composition of the experimental diet used in Fig. 3-4 is summarized. Abbreviations: BCAA, branched-chain amino acid.

**Supplementary Table S4-4. Composition of the experimental diet (BCAA diet)**

| Composition (%) | Group  HPi  + BCAA_0 | Group  HPi  + BCAA_10 |
| --- | --- | --- |
| Casein | 10.8 | 10.8 |
| Egg albumin | 0 | 0 |
| BCAA | 0 | 10.0 |
| Starch | 26.4 | 16.4 |
| KH_2_PO_4_ | 5.6 | 5.6 |
| TD05030 | 57.2 | 57.2 |

Composition of the experimental diet used in Fig. 5-6 is summarized. Abbreviations: BCAA, branched-chain amino acid. Ratio of valine: leucine: isoleucine in the BCAA was 1:2:1.

**Supplementary Table S5. Primer Nucleotide Sequences**

|  | Forward | Reverse |
| --- | --- | --- |
| *Havcr1* | 5’- AGACTGGAATGGCACTGTGA -3’ | 5’- GGCTTCCTCAAAGGGATTCT -3’ |
| *Tnf* | 5’- AAATGGCAAATCGGCTGACG -3’ | 5’- GTAGCCCACGTCGTAGCAAA -3’ |
| *Icam1* | 5’- GTGCACCTGACAGTGCTGTACCA -3’ | 5’- CCACGATCACGAAGCCCGCA-3’ |
| *Tgfb1* | 5’- TGCTTCAGCTCCACAGAGAA -3’ | 5’- TGGTTGTAGAGGGCAAGGAC -3’ |
| *Col1a1* | 5’- CCAGCTGACCTTCCTGCGCC -3’ | 5’- TCCGGTGTGACTCGTGCAGC -3’ |
| *Ppm1k* | 5’- AGTTATGCCCACCTGTCTGC -3’ | 5’- ACCATCTCTCAACAGGGCTACT -3’ |
| *Gapdh* | 5’- GGATGCAGGGATGATGTT -3’ | 5’- TGCACCACCAAGTGCTTA -3’ |

Abbreviations: *Havcr1*, hepatitis A virus cellular receptor 1 encoding kidney injury molecule 1 (KIM1); *Tnf,* tumor necrosis factor; *Icam1,* intercellular adhesion molecule 1; *Tgfb1*, transforming growth factor β1; *Col1a1*, collagen type 1 alpha 1 chain; *Ppm1k*, protein phosphatase 1k encoding protein phosphatase 2C family member (PP2Cm); *Gapdh*, glyceraldehyde-3-phosphate dehydrogenase. The primers sequences were designed according to the sequence of each gene deposited in the GenBank database.

**Supplementary Figure S1.**


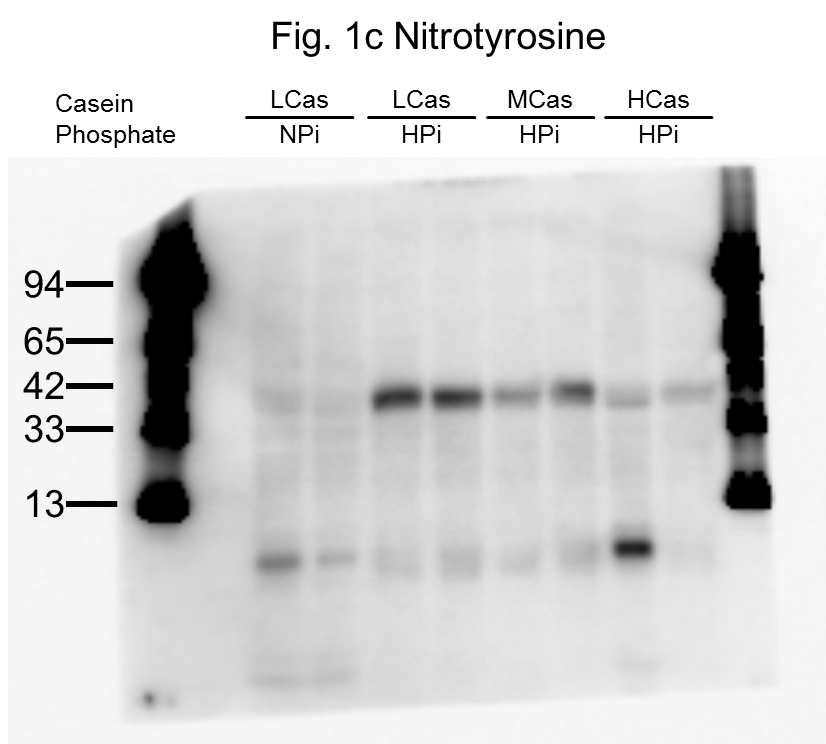


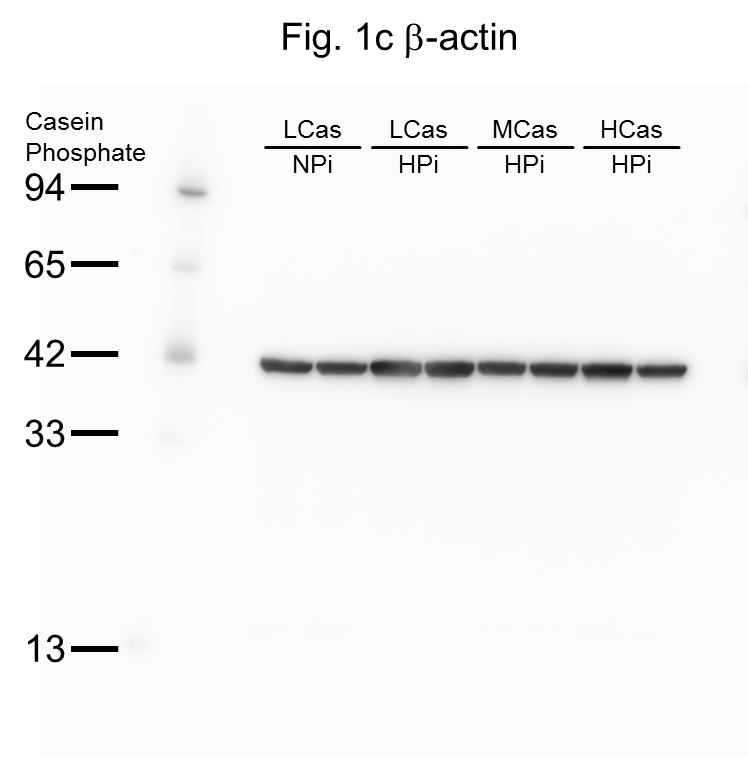


Full blots of Fig. 1c are shown.

**Supplementary Figure S2.**


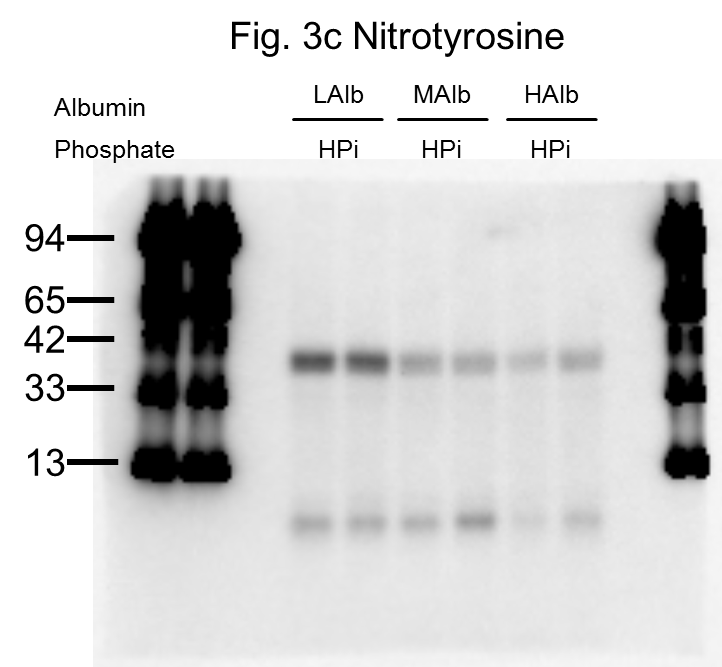


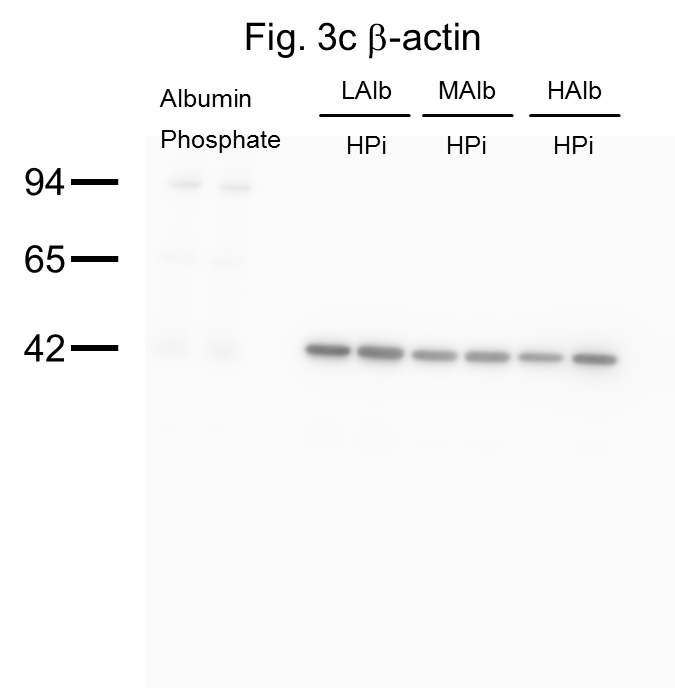


Full blots of Fig. 3c are shown.

**Supplementary Figure S3.**


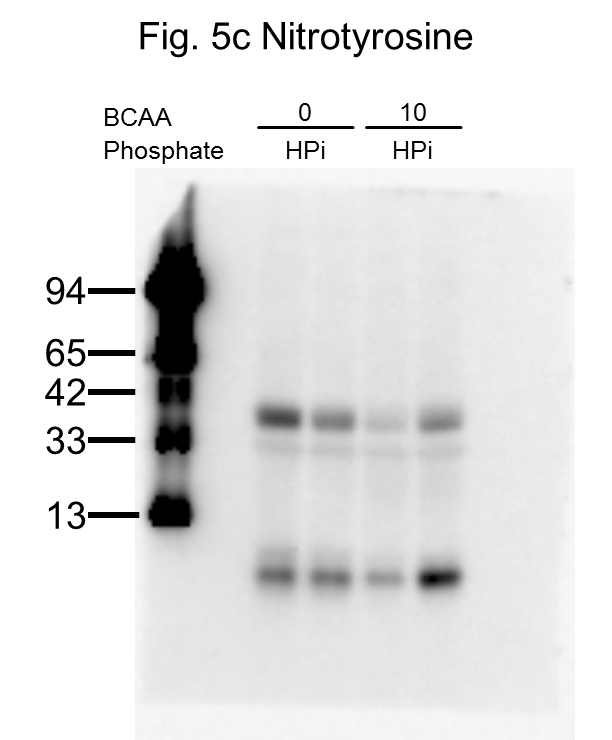


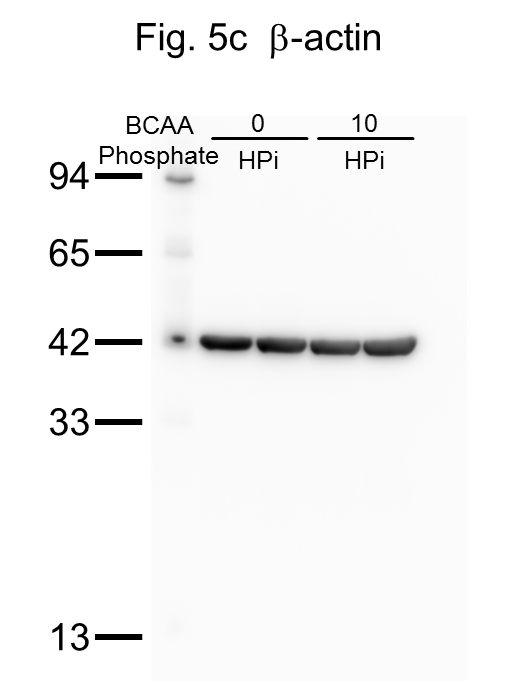


Full blots of Fig. 5c are shown.

**Supplementary Figure S4.**


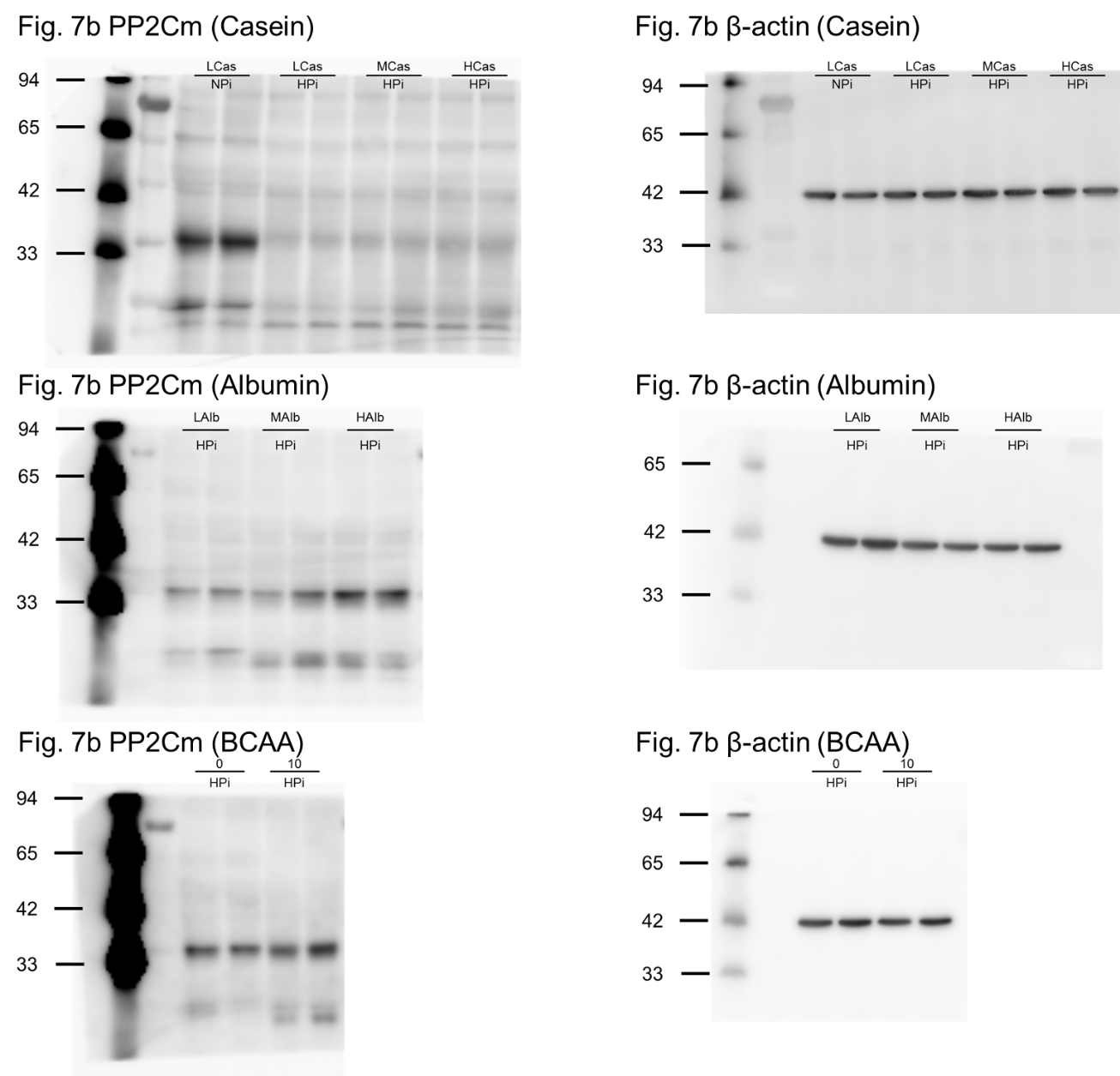


Full blots of Fig. 7b are shown
